# Evaluation of modified and newly applied patella height indices in primary total knee arthroplasty

**DOI:** 10.1007/s00256-022-04142-1

**Published:** 2022-08-09

**Authors:** Anna Janine Schreiner, Lena Spiegel, Shuang Gen Yan, Christian Konrads, Felix Erne, Philipp Hemmann, Florian Schmidutz

**Affiliations:** 1 Orthopaedic Clinic Markgroeningen, Markgroeningen, Germany; 2 Department of Traumatology and Reconstructive Surgery, BG Unfallklinik Tuebingen, Eberhard Karls University of Tuebingen, Tuebingen, Germany; 3 Department of Orthopaedic Surgery, The First Affiliated Hospital of Anhui Medical University, No. 1 Baicao Road, Hefei, 230088 China; 4 Department of Orthopaedic Surgery, University of Tuebingen, Tuebingen, Germany; 5 Department of Orthopaedic Surgery, Physical Medicine and Rehabilitation, University of Munich (LMU), Munich, Germany; 6 Orthopedic Surgery, Orthopedic Center Rosenheim, Rosenheim, Germany

**Keywords:** Patella height, Patella height index, Primary total knee arthroplasty, Patella alta, Patella baja

## Abstract

**Objective:**

The aim of this radiological study was to compare several relevant modified and newly applied patella height indices (PHI) in navigated primary total knee arthroplasty (TKA) to determine intra- and interobserver reliability in order to give a recommendation for clinical application in measuring patella height (PH) in primary TKA.

**Materials and methods:**

A retrospective data analysis assessing different PHI (modified Insall-Salvati index (mISI), Caton-Deschamps index (mCDI), Blackburne-Peel index (mBPI), Plateau-Patella Angle (mPPA); Miura-Kawaramura index (MKI), Knee-Triangular index (KTI)) on lateral knee radiographs was performed by two blinded observers using the same software three months pre- and postoperatively. Concordance correlation coefficient and Pearson’s correlation respectively were determined for intra- and interobserver rating as well as a categorization according to Landis and Koch and Cohen.

**Results:**

A total of 337/291 patients of a 5-year period could be analyzed pre-/postoperatively. Excellent postoperative interrater results according to the categorization of Landis and Koch were achieved for the mBPI (Pearson 0.98) > mPPA (0.90) > KTI (0.86), good results for the MKI (0.79) and the mCDI (0.69), and moderate results for the mISI (0.52) with a predominantly strong Cohen correlation in almost all cases. Preoperatively, the mBPI and the KTI were the best interrated PHI. No PH changes could be found postoperatively for the mISI, KTI, MKI, and mPPA.

**Conclusion:**

The mBPI, the mPPA, and the KTI can be recommended for PH assessment in TKA. The mPPA might be the easiest one to use in a daily clinical set-up.

## Introduction

Measuring patella height (PH) has been a topic of debate for decades and there are multiple different radiological indices available for native knees. Patella infera (also referred to as patella baja) is defined as a direct shortening of the patellar tendon whereas pseudo patella infera results from a relative or indirect alteration of the PH, e.g., due to joint line elevation after total knee arthroplasty (TKA) without alteration of the length of the patellar ligament. Patella infera can be a combination of true PH alteration determined by the length of the patellar ligament as well as pseudo-PH alteration. In contrast, patella alta is an abnormally high patella in relation to the femur. The most common and clinically applied methods for determining PH in native knees are the indices or ratios respectively according to Insall Salvati, Blackburne-Peel, and Caton-Deschamps [1–3]. In general, these methods are based on the analysis of lateral X-ray images of the knee joint setting in relation a distance of the patella (patella joint surface or diagonal diameter of the patella) and a distance from a point at the patella to a reference point on the tibia and/or femur under avoidance of a radiological magnification error. Different patella height indices (PHI) are each associated with certain limitations often refer to different and not always comparable reference landmarks. Furthermore, not all PHI are suitable for knees with implanted endoprosthesis which is why some authors developed modified patella height indices (mPHI) with altered and TKA components’ referenced landmarks for the application in knees with TKA. As PH alterations are under discussion as one reason for impaired clinical outcomes after TKA, it is important to evaluate whether PHI and mPHI are useful analysis tools for this concern also given the fact that valid measuring and comparing of PH is still a challenging issue with no gold-standard yet. Besides, not all clinically relevant scores have been profoundly evaluated so far with regard to intra- and interobserver reliability and there is no sufficient data available especially for PHI in TKA which seems to be even more challenging with regards to proper evaluation.

Therefore, the aim of this retrospective radiological study was to compare several relevant modified and newly applied patella height indices pre- and postoperatively in navigated primary TKA to determine intra- and interobserver reliabilities in order to give a recommendation for clinical application in measuring PH in primary TKA.

## Materials and methods

A retrospective data analysis assessing different PHI consisting of digital pre- and postoperative knee radiographs was performed by two blinded with the same software tool. There was no pre-selection. The data was digitally recorded on an electronic picture archiving and communications system (PACS). All radiographs were measured by both observers separately with the second measurement made 3 months later (intraobserver assessment) and compared against each other (interobserver assessment). Before, we had retrospectively identified all patients of a 5-year period from our hospital database who received a primary bicondylar, posterior cruciate retaining cemented standard TKA implanted in a navigated manner in tibia-first-technique by the same surgeon consultant. The study was approved by the local ethics committee (no. 550/2017BO2) prior to review. Written informed consent was not required. The study was done in agreement with the ethical standards of the institutional and national research committee and with the 1964 Helsinki Declaration and its later amendments respectively comparable ethical standards.

### General data

The total study cohort consisted of 396 patients (male/female *n* = 167/229) with an average age of 66 ± 10 (41–93) years at the time of surgery. Eighty-five of all patients were obese. Two hundred ten right and 186 left knees received TKAs. Indications for TKA were primary osteoarthritis (*n* = 265), posttraumatic osteoarthritis (*n* = 77), and secondary osteoarthritis (*n* = 54).

All patients underwent navigated primary total knee arthroplasty performed by the same experienced consultant orthopaedic surgeon using the same implant (B. Braun Aesculap, Columbus®) strictly according to internal standard operation procedures in a navigated manner based on infrared technology (B. Braun Aesculap, OrthoPilot®). After median skin incision, the Hoffa fat pad was partially resected after anteromedial arthrotomy was performed. Patellar denervation and resection of patellar osteophytes was conducted in all cases. Patella resurfacing was only performed in severe cases of retropatellar cartilage damage. After determination of the leg axis, the tibia cut was made followed by ligament-based preparation of the distal femur. Prior to the cemented implantation of the tibial and femoral component, the pneumatic tourniquet was applied followed by jet lavage of the bony surface. In addition to navigation control, range of motion, knee stability, and patella tracking were manually controlled. Regular wound closure was performed. All patients were mobilized with allowed full-weight bearing at the first day after surgery and received physiotherapy on a daily basis until submission to a rehabilitation center.

Besides radiographic parameters, pre- and postoperative range of motion, the use of ultra-congruent (UC) inlays, patellar resurfacing, and ipsilateral THA were analyzed as well.

### General radiographical analysis

Lateral knee as well as a.p. leg axis radiographs were taken 3 months pre- and postoperatively as well as intraoperatively and during the hospital stay. All radiographs within 3 months pre- and postoperatively were reviewed in a retrospective manner by two blinded and independent observers (LS, SGY) with different levels of orthopedic training using the same tool as for TKA planning (mediCAD, Hectec Gmbh; Landshut, Germany). Measurements for PHI analysis were made on the lateral radiograph with the knee in at least 30 degrees of flexion. Radiographs were excluded if the measurements could not be accurately determined by the raters due to flexion, rotation, or other insufficiencies. A total of 337/291 radiographs out of 396 each could be analyzed pre-/postoperatively corresponding to an exclusion rate of *n* = 59/105 pre-/postoperative radiographs.

Besides evaluation of patella norma/alta/infera and the PHI as described below, the parameters mechanical leg axis and mechanical joint angles (mLDFA, mMPTA) were analyzed on a.p. standing total leg radiographs.

### Patella height indices

The following known and modified radiological methods for PH analysis in knees with TKA on the lateral knee joint radiographs were chosen and evaluated:

- modified Insall-Salvati index (mISI) [4]
- modified Caton-Deschamps index(mCDI)[5]
- modified Blackburne-Peel index(mBPI)[6]
- modified Plateau-Patella Angle(mPPA)[7]

Furthermore, the following newly applied PHI were also evaluated with regard to their applicability in TKA:

- Miura-Kawamura index (MKI)[8, 9]
- Knee-Triangular-index(KTI)[10].

**Fig. 1:**
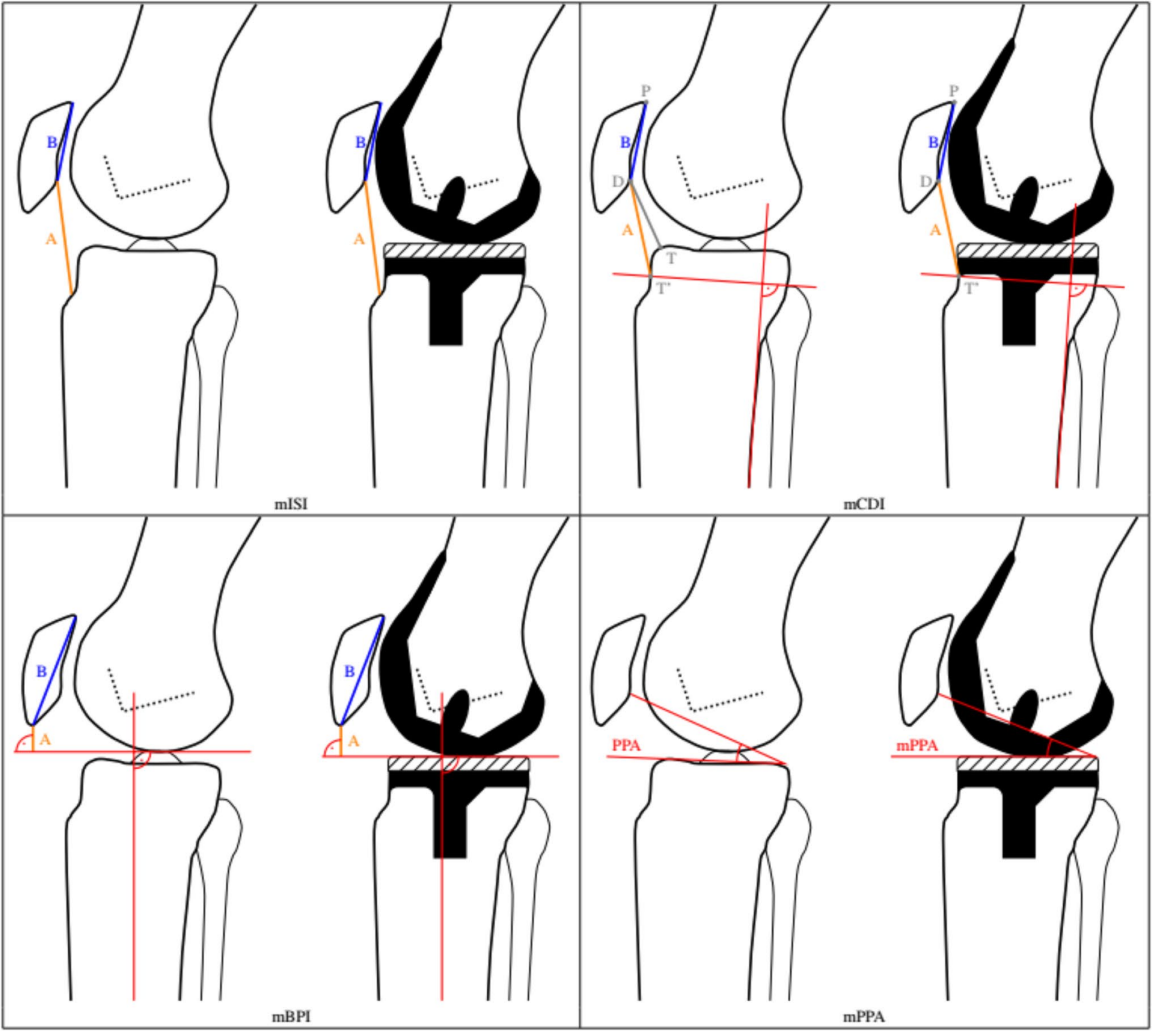
The technical measurements on lateral knee radiographs for the following mPHI are displayed: Modified Insall-Salvati index (mISI), modified Caton-Deschamps index (mCDI), modified Blackburne-Peel index (mBPI), modified Plateau-Patella Angle (mPPA)

**Fig. 2:**
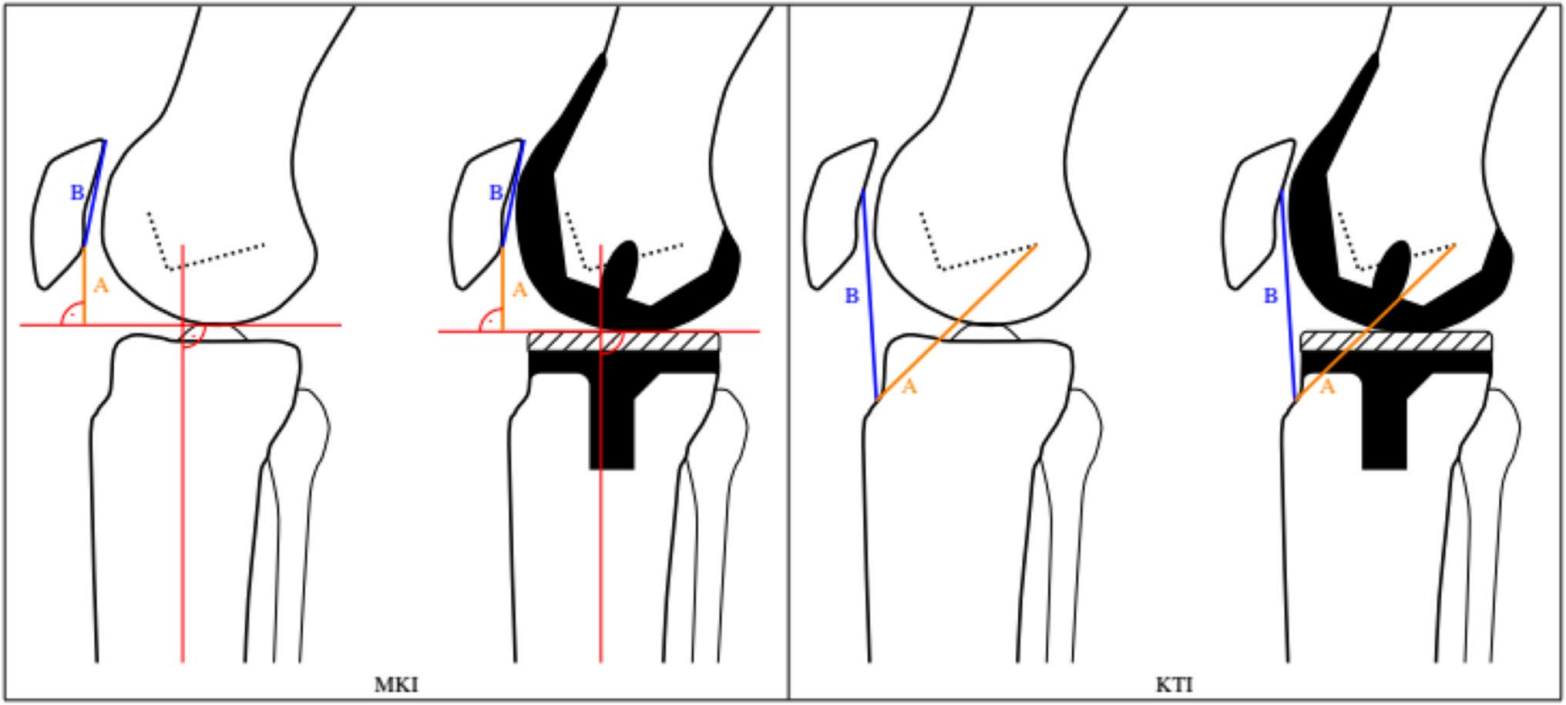
The technical measurements on lateral knee radiographs for the following new PHI are displayed: Miura-Kawaramura index (MKI), Knee-Triangular index (KTI)

All indices and angles and their measurements respectively are displayed and summed up in Figs. 1 and 2. While the indices are usually displayed as sole numbers as a result of a ratio, the mPPA is displayed in degrees.

### Statistical methods

For statistical analysis, Microsoft Excel (office 2016) was used. Descriptive statistics were used to describe the baseline characteristics of the study population and the PHI analysis. Mean values are presented with ± standard deviation (minimum–maximum). Absolute and relative differences in PH changes were identified as well. Intraobserver reproducibility was assessed using the concordance correlation coefficient, comparing two sets of measurements done by every observer for the whole cohort, in a blinded manner. Interobserver agreement was also assessed using the concordance correlation coefficient to compare measurements of the same knee radiographs by the two observers who independently made their measurements and who were blinded to each other’s assessment. Besides, the confidence interval was determined and the validity of methods was evaluated by comparing them against each other using Pearson’s correlation (P) coefficient. In addition, Cohen correlation (CC) was assessed to determine the strength or effect size respectively of the measured correlations with regards to the interobserver reliability as well as the evaluation according to Landis and Koch (LK) to determine and categorize the observers’ agreement. R =  ± 0.1/0.30/0.50 (translated to 1/2/3) stands for a small/medium/large effect size and a kappa value of < 0.20/0.21–0.40/0.41–0.60/0.61–0.80/0.81–1.00 (displayed as 1–5) corresponds to a poor/weak/moderate/good/very good result. Normal distribution was checked and confirmed. The level of statistical significance is reported as *p*-value (*p* < 0.05). The study and its statistical analysis was performed according to the STARD initiative for studies of diagnostic accuracy [11].

## Results

### General data and general radiographic analysis

The mean values for leg axis analysis and mechanical joint angles are presented in Table 1 showing that TKA successfully corrects a.p. varus > valgus deformities and pathological a.p. angles. A UC inlay was used in 48 cases and patella resurfacing was performed in 10 patients. Patella resurfacing did not affect PH measurements in these few cases relative to those individuals who did not undergo this procedure as it was performed without significantly changing the diameter and length of the patella which was checked by comparing the preoperative radiographs. Resection of the osteophytes and denervation were the main part of the procedure. Twenty-six patients had an ipsilateral total hip arthroplasty. Average range of motion for extension/flexion was 0–5-110° 3 months pre- and 0–0-100° postoperatively.

**Table 1:** Mechanical leg axis and mechanical joint angles pre- and postoperatively after navigated primary TKA 3 months pre- and postoperatively; values are presented with SD and min–max

	Mechanical leg axis	Varus Valgus	mLDFA	90° deviation from the LDFA	mMPTA	90° deviation from the MPTA
Preop	− 3.8 ± 6.9 (− 20.8–17.3)	*n* = 290 *n* = 104	88.1 ± 2.8 (79.1–98.2)	2.7 ± 2.0 (0.1–10.9)	87.0 ± 3.5 (78.3–96.4)	3.8 ± 2.5 (0.0–11.7)
Postop	− 1.8 ± 2.2 (− 8.6–4.6)	*n* = 265 *n* = 65	90.9 ± 1.7 (83.5–95.4)	1.6 ± 1.1 (0.0–6.5)	89.1 ± (1.6) (84.1–94.7)	1.4 ± 1.1 (0.0–5.9)

*preop.* preoperatively, *postop*. postoperatively, *mLDFA* mechanical lateral distal femur angle, *mMPTA* mechanical proximal tibial angle, *SD* standard deviation, *min* minimum, *max* maximum

### Patella height indices data

The mean values for all evaluated PHI pre- and postoperatively generated by both observers are compiled in Table 2. This and its values respectively can serve as a current reference with average mean values for all evaluated PHI pre- and postoperatively for clinicians and scientists analyzing PH changes in patients with TKA. Subdivided into results of normal PH, patella alta, and infera (corresponding to the different given value ranges according to the different PHI and their literature), no changes could be found postoperatively for mISI (1.25), KTI (1.0 ± 0.1), MKI (no delta > 10%) and mPPA (21–29%), whereas patella alta resulted according to measurements with the mCD (0.6–1.2) and infera according to the mBPI (0.54–1.06).

**Table 2:** Average mean values for all evaluated PHI pre- and postoperatively generated by both blinded observers; values are presented with SD and min–max

	mISI	mCDI	mBPI	mPPA	MKI	KTI
**Preop**	**1.7 ± 0.19** **(1.16–2.55)**	**1.36 ± 0.2** **(0.77–2.48)**	**0.47 ± 0.14** **(0.04–1.07)**	**25.1 ± 3.93** **(12.2–41.2)**	**0.93 ± 0.15** **(0.54–1.64)**	**1.0 ± 0.07** **(0.81–1.11)**
Observer 1	1.71 ± 0.17 (1.24–2.38)	1.34 ± 0.17 (0.84–2.07)	0.46 ± 0.14 (0.04–1.05)	25.15 ± 3.77 (17.1–38.5)	0.91 ± 0.14 (0.54–1.5)	1.01 ± 0.07 (0.82–1.22)
Observer 2	1.69 ± 0.22 (1.16–2.55)	1.37 ± 0.22 (0.77–2.48)	0.47 ± 0.15 (0.06–1.07)	25.05 ± 4.1 (12.2–41.2)	0.95 ± 0.16 (0.57–1.64)	0.98 ± 0.07 (0.81–1.18)
**Postop**	**1.71 ± 0.19** **(0.88–2.74)**	**1.34 ± 0.19** **(0.39–2.53)**	**0.42 ± 0.15** **(0.02–1.11)**	**27.61 ± 3.98** **(14.0–42.5)**	**0.86 ± 0.15** **(0.41–1.74)**	**1.03 ± 0.08** **(0.78–1.37)**
Observer 1	1.7 ± 0.16 (1.14–2.43)	1.31 ± 0.17 (0.81–2.11)	0.41 ± 0.14 (0.02–1.05)	27.42 ± 3.87 (14.0–42.0)	0.84 ± 0.14 (0.41–1.54)	1.04 ± 0.07 (0.84–1.37)
Observer 2	1.72 ± 0.22 (0.88–2.74)	1.37 ± 0.21 (0.39–2.53)	0.42 ± 0.15 (0.03–1.11)	27,83 ± 4.08 (17.2–42.5)	0.88 ± 0.16 (0.46–1.74)	1.02 ± 0.08 (0.78–1.36)
**Absolute pre-post difference**	+ 0.01	− 0.01	− 0.05	+ 2.51	− 0.07	+ 0.03
**Relative pre-post** **difference (%)**	0.46	− 1.07	− 12.6	9.08	− 1.08	3.26

*preop*. preoperatively, *postop*. postoperatively, *mISI* modified Insall-Salvati-Index, *mCDI* modified Caton-Deschamps index, *mBPI* modified Blackburne-Peel index, *mPPA* modified Plateau-Patella Angle, *MKI* Miura-Kawamura index, *KTI* Knee-Triangular-index

Intraobserver data is presented in Tables 3 and 4 and interobserver results are summed up in Table 5. In summary, all measured PHI showed good intraobserver results pre- and postoperatively for observer 1 (CC, LK), whereas observer 2 demonstrated good results only for the mBPI and the mPPA respectively. Excellent postoperative interrater results according to the categorization of Landis and Koch were achieved for the mBPI (Pearson 0.98) > mPPA (0.90) > KTI (0.86), good results for the MKI (0.79) and the mCDI (0.69), and moderate results for the mISI (0.52) with a strong Cohen correlation (3) in almost all cases. Preoperatively, the mBPI and the KTI were the best interrated PHI. The mISI showed the worst intra- and interrater results over almost all categories/observers.

**Table 3:** Pre- and postoperative intraobserver rating (observer 1)

	mISI pre T1/T2	mCDI pre T1/T2	mBPI pre T1/T2	mPPA preT1/T2	MKI pre T1/T2	KTI pre T1/T2
** CC**	**0.95**	**0.98**	**0.99**	**0.95**	**0.98**	**0.98**
CI	0.03	0.03	0.03	0.72	0.02	0.01
SD	0.16	0.2	0.17	4,48	0.15	0.08
Range	150	150	150	150	150	150
From	0.93	0.95	0.96	0.23	0.95	0.97
To	0.98	1.01	1.02	1.67	1.0	1.00
T test	0.00	0.00	0.39	0.23	0.00	0.00
** LK**	**5**	**5**	**5**	**5**	**5**	**5**
Cohen	3	3	3	3	3	3
	mISI post T1/T2	mCDI post T1/T2	mBPI post T1/T2	mPPA post T1/T2	MKI post T1/T2	KTI post T1/T2
** CC**	**0.95**	**0.97**	**0.99**	**0.99**	**0.99**	**0.98**
CI	0.03	0.04	0.03	0.75	0.03	0.01
SD	0.18	0.22	0.18	4.7	0.18	0.08
Range	150	150	150	150	150	150
From	0.92	0.94	0.96	0.24	0.96	0.97
To	0.98	1.01	1.02	1.75	1.01	1.00
T test	0.00	0.00	0.00	0.1	0.00	0.26
** LK**	**5**	**5**	**5**	**5**	**5**	**5**
Cohen	3	3	3	3	3	3

*pre* preoperatively, *post* postoperatively, *T1/2* 3 months pre/post, *mISI* modified Insall-Salvati-Index, *mCDI* modified Caton-Deschamps index, *mBPI* modified Blackburne-Peel index, *mPPA* modified Plateau-Patella Angle, *MKI* Miura-Kawamura index, *KTI* Knee-Triangular-index; *CC* concordance correlation, *CI* confidence interval, *SD* standard deviation, *LK* Landis and Koch, *Cohen* Cohen correlation

**Table 4:** Pre- and postoperative intraobserver rating (observer 2)

	mISI pre T1/T2	mCDI pre T1/T2	mBPI pre T1/T2	mPPA preT1/T2	MKI pre T1/T2	KTI pre T1/T2
** CC**	**0.46**	**0.61**	**0.96**	**0.64**	**0.57**	**0.63**
CI	0.03	0.03	0.13	3.95	0.02	0.01
SD	0.19	0.19	0.13	3.95	0.14	0.07
Range	130	130	130	130	130	130
From	0.42	0.58	0.94	-0.03	0.55	0.62
To	0.49	0.64	0.99	1.32	0.59	0.64
T test	0.00	0.00	0.00	0.15	0.00	0.00
** LK**	**3**	**4**	**5**	**4**	**3**	**4**
Cohen	3	3	3	3	3	3
	mISI post T1/T2	mCDI post T1/T2	mBPI post T1/T2	mPPA post T1/T2	MKI post T1/T2	KTI post T1/T2
** CC**	**0.3**	**0.37**	**0.96**	**0.85**	**0.72**	**0.74**
CI	0.04	0.04	0.02	0.76	0.03	0.01
SD	0.21	0.21	0.13	4.04	0.16	0.08
Range	112	112	112	112	112	112
From	0.26	0.34	0.94	0.1	0.69	0.72
To	0.34	0.41	0.98	1.6	0.75	0.75
T test	0.00	0.00	0.05	0.14	0.00	0.00
** LK**	**2**	**2**	**5**	**5**	**4**	**4**
Cohen	2	2	3	3	3	3

*pre* preoperatively, *post* postoperatively, *T1/2* 3 months pre/post, *mISI* modified Insall-Salvati-Index, *mCDI* modified Caton-Deschamps index, *mBPI* modified Blackburne-Peel index, *mPPA* modified Plateau-Patella Angle, *MKI* Miura-Kawamura index, *KTI* Knee-Triangular-index, *CC* concordance correlation, *CI* confidence interval, *SD* standard deviation, *LK* Landis and Koch, *Cohen* Cohen correlation

**Table 5:** Interobserver correlations according to mean PHI average values of both raters (observer 1 and 2, see also Tables 3 and 4)

	mISI pre	mCDI pre	mBPI pre	mPPA	MKI pre	KTI pre
CC	0.52	0.71	0.98	0.78	0.73	0.86
CI	0.02	0.02	0.01	0.31	0.01	0.01
SD	0.02	0.20	0.14	3.93	0.15	0.07
Range	630	630	630	630	630	630
From	0.53	0.72	0.99	1.08	0.74	0.87
To	0.50	0.69	0.96	0.47	0.72	0.85
T test	0.03	0.00	0.00	0.17	0.00	0.00
** Pearson**	**0.52**	**0.71**	**0.98**	**0.78**	**0.73**	**0.86**
** LK**	**3**	**4**	**5**	**4**	**4**	**5**
Cohen	3	3	3	3	3	3
	mISI post	mCDI post	mBPI post	mPPA post	MKI post	KTI post
CC	0.56	0.69	0.98	0.90	0.79	0.86
CI	0.02	0.02	0.01	0.33	0.01	0.01
SD	0.19	0.19	0.15	3.98	0.15	0.08
Range	550	550	550	551	550	550
From	0.58	0.71	0.99	1.33	0.81	0.87
To	0.55	0.68	0.97	0.57	0.78	0.85
T test	0.02	0.00	0.00	0.18	0.00	0.00
** Pearson**	**0.56**	**0.69**	**0.98**	**0.90**	**0.79**	**0.86**
** LK**	**3**	**4**	**5**	**5**	**4**	**5**
Cohen	3	3	3	3	3	3

*pre* preoperatively, *post* postoperatively, *mISI* modified Insall-Salvati-Index, *mCDI* modified Caton-Deschamps index, *mBPI* modified Blackburne-Peel index, *mPPA* modified Plateau-Patella Angle, *MKI* Miura-Kawamura index, *KTI* Knee-Triangular-index, *CC* concordance correlation, *CI* confidence interval, *SD* standard deviation, *Pearson* Pearson correlation, *LK* Landis and Koch, *Cohen* Cohen correlation

## Discussion

Patella height can change after TKA and is often reported to be lowered after surgery which can then lead to patellar tendon rupture, impingement and anterior knee pain, decreased range of motion, extensor lag, and a decreased lever arm [12–14]. Patellofemoral symptoms in general have been shown to be responsible for a large percentage of revisions of TKAs and PH alterations represent poor prognostic indicators in TKA [7, 15–17]. Gaillard et al. report on the influence on PH on TKA showing that clinical and radiological results for TKAs with pre-operative patella alta and patella baja were comparable to TKAs with a normal pre-operative patellar height, but the risk of post-operative patellar fracture increased for patients with pre-operative patella baja [18]. Dos-Santos et al. additionally confirm that pseudo-patella baja has a clinical relevance that should not be overlooked as patients often complain about anterior knee pain and a higher frequency of flexion contracture [19]. This context displays the need of a reliable single method or combination of methods to easily and validly determine PH in TKA. Determining the most accurate and precise way to measure PH in general and especially before and after TKA has been a controversial debate so far and many different measurements and ratios have been applied each associated with different or significant limitations [7, 20–22]. Most current methods need two measurements and one calculation which can be time consuming also considering the normal ranges which have to be remembered. A new PH measuring method by Portner et al. bypasses this problem by using an angle measurement [7, 23, 24]. To address the measurement problems in patients with TKA resulting from applying standard PHI like the well-known Caton-Deschamps (CD), Insall-Salvati (IS) or Blackburne-Peel (BP) index in native knees, modified PHI have been developed and further new PH measurement options emerged as well. For both, modified and new options, none or insufficient comparing literature data regarding their application, validation, and reliability in TKA patients are available. For the first time and in a large scale, this study therefore retrospectively evaluated several relevant modified and newly applied patella height indices pre- and postoperatively in navigated primary TKA to provide extensive data for these PHI as well as to determine their intra- and interobserver reliability in order to give a recommendation for clinical application in measuring PH in primary TKA. In summary, the present study demonstrated that the mBPI, mPPA, and KTI show the best intra- and interrater results pre- and postoperatively for PHI assessments. Furthermore, we assessed data regarding PH analysis with mPHI and newly applied PHI in TKA which has not been available in the current literature until now. To our best knowledge, this is the first study for (m)PHI evaluating and comparing so many of these special indices. We could present a valid table which can serve as a current reference with average mean values for all evaluated PHI pre- and postoperatively for clinicians and scientists analyzing PH changes in patients with TKA. This is something new for new PHI as well as mPHI and might be also useful for further studies for continued comparison and other comprehensive evaluations. Compared to norm ranges known or evaluated so far, the majority of TKAs showed no pathological PH values. Yet, patella alta measurements resulted according to mCDI analysis and -infera results according to the mBPI with the latter not being quite far from the norm range which correlates with the current literature. Furthermore, several mPHI and one of the new PHI can serve as good options for assessing osteoarthritic knees and those after TKA. Interestingly, observer 1 showed very good results according to CC and LK for all six methods, whereas observer 2 only demonstrated good intrarater agreements for two PH measurement options. This indicates that most of the PH assessing methods require precision, time, and concentration which probably do not make all of them applicable in daily clinical routine but they still might be useful for special academic questions. Observer 2 and the interobserver assessment show that that the mBPI, mPPA and KTI can therefore be recommend for both set-ups with the angle measurement of the mPPA probably being the easiest one to apply in daily clinical routine. We could also present further TKA data of mechanical leg axis and mechanical joint angles after navigated primary TKA 3 months pre- and postoperatively demonstrating that TKA corrects a.p. varus > valgus deformities and pathological a.p. angles. So, there is potential for more evenly distributed load transfer after TKA according to the philosophy of mechanical alignment. Our patients also lost their preoperative extension deficit after surgery and as there were only little cases with retropatellar cartilage damage, patellar resurfacing was only necessary in 10 cases.

Compared to the little adequate literature available, we offer new data for (m)PHI in TKA. Xu et al. evaluated standard PHI and the mISI and demonstrated an increase in (pseudo) patella baja after TKA with greater importance for this diagnosis using the indices of CD and BP; they also showed that the mISI is superior to the classic Insall-Salvati index [25]. Konrads et al. evaluated the use of the derived CDI versus the original CD amongst others and demonstrated high inter- and interrater agreements [26]. Yet, in comparison to our many evaluated mPHI, the mCDI as well as the mISI did not range amongst the (m)PH measurement options with the best inter- and intrarater results, and even though Cabral et al. demonstrated reproducible results for the most common three original methods of CD/BP/IS, these old methods should no longer be applied at least for patients after TKA because valid and better options like the mBPI or mPPA are available as shown with our data [27]. In our case, the mISI showed the least good results compared to all other PH measurement techniques and Rogers et al. also say that the theoretical advantage of using the Insall-Salvati and modified Insall-Salvati ratios in measuring true patellar height after total knee arthroplasty needs to be balanced against their significant interobserver variability and inferior reliability when compared with other ratios [21]. Konrads et al. evaluated the derived CDI and the mBPI amongst other original PHI and could show good intra- and interobserver agreements in comparison to the original indices [28]. They could also state that patella infera was not correlated with reduced subjective or objective clinical outcome parameters when PH was altered not more than 10%. We can confirm good results for the use of the mBPI as well. The mCDI should not be used because there are better options for mPHI like the mPPA or the new PHI KTI. We consider the mPPA to me the most practical and robust PHI. It was evaluated in several steps in native, osteoarthritic and TKA knees always demonstrating a very good reproducibility and reliability as well as independency from rater experience [7, 23, 24]. It does not require a calculation based on the measurement of different lengths or diameters but only the measurement of an angle which is much easier to perform in a daily clinical set-up, rapidly with a goniometer, e.g., or digital software. Besides, the range is easy to remember. This angle furthermore can better prevent mis-analysis of PH in cases of knees with previous surgeries, e.g., as well as in general as it does not use the length of the patella or its tendon, but rather relates the patella to the joint line similar to the Blackburne-Peel method. In addition, the influence of the radiological setting in determining PHI is emphasized by the study of Pfitzner et al. [29] which underlines the importance of easily to apply PH measurement techniques.

Our study is limited by a retrospective study design and a small rater group. Besides, larger cohorts and the comparison of different surgeons might reveal more additive information for a more detailed comparison of different PHI. More clinical studies with a correlation of (m)PHI and clinical scores will be crucial for determining the clinical use of the different PHI.

Furthermore, our study cohort includes patients with primary and secondary osteoarthritis of which *n* = 30 of the latter were patients with secondary osteoarthritis after HTO. HTO is a procedure which can also lead to changes in sagittal or coronar anatomy like slope or PH deviations, e.g., but there are also publications saying that HTO does not affect PH [30, 31]. It additionally seems to be the case that also depends on the HTO technique whether PH alterations might occur less likely or not [30, 32]. Nevertheless, the surgeon has to perform TKA correctly no matter if the patient’s osteoarthritis is primary or secondary and all forms of osteoarthritis can present with pre-existent PH alterations either to primary or posttraumatic or after-HTO osteoarthritis. This is why we included all cases of osteoarthritis cases in our study as our focus lies on the application of PHI in TKA knees.

In summary, our study showed that assessing PH requires accuracy and can then lead to overall good interrater results. With respect to all our intra- and interobserver data, we advise to apply the measurement of PH in osteoarthritic native knees as well as after TKA with the mBPI and the mPPA as well as the new method KTI for academic and scientific as well as clinical PH analysis. Regarding applicability and feasibility, the mPPA might be the easiest one to use in a daily clinical set-up as it only requires the measurement of an angle and makes calculation based on two different measurements dispensable.

## Declarations

Approval from the Institutional Review Board was obtained, and in keeping with the policies for a retrospective analysis, informed consent was not required.
